# Dr. Leonard Strong McManus

**Published:** 1911-04-01

**Authors:** 


Dr. Leonard Strong McManus has died in London at the age of fifty-one. He qualified as M.D. and M.Ch. at the Royal University of Ireland, and came to Battersea about twenty-five years ago, where he was engaged very usefully in municipal work. He sat as a Moderate on the borough council since 1894, and originated the borough milk depôt, the first of its kind in London. A presentation of a cheque and two pieces of plate was made to him quite recently. He had been surgeon to the Bolingbroke Hospital and Dispensary, of which he was afterwards a governor. He was connected with many medical and hygienic societies.

